# Study protocol: fit for delivery - can a lifestyle intervention in pregnancy result in measurable health benefits for mothers and newborns? A randomized controlled trial

**DOI:** 10.1186/1471-2458-13-132

**Published:** 2013-02-13

**Authors:** Linda Reme Sagedal, Nina C Øverby, Hilde Lohne-Seiler, Elling Bere, Monica K Torstveit, Tore Henriksen, Ingvild Vistad

**Affiliations:** 1 Department of Obstetrics and Gynecology, Sorlandet Hospital, Kristiansand, Norway; 2 Department of Public Health, Sports and Nutrition, University of Agder, Kristiansand, Norway; 3 Research Department, Sorlandet Hospital, Kristiansand, Norway; 4 Department of Obstetrics and Gynecology, Oslo University Hospital, Oslo, Norway

**Keywords:** Pregnancy, Gestational weight gain, Nutrition, Exercise, Large for gestational age, Gestational diabetes, Weight retention, Randomized controlled trial

## Abstract

**Background:**

The global obesity epidemic has led to increased attention on pregnancy, a period when women are at risk of gaining excessive weight. Excessive gestational weight gain is associated with numerous complications, for both mother and child. Though the problem is widespread, few studies have examined the effect of a lifestyle intervention in pregnancy designed to limit maternal weight gain. The Fit for Delivery study will explore the effectiveness of nutritional counseling coupled with exercise classes compared with standard prenatal care. The aims of the study are to examine the effect of the intervention on maternal weight gain, newborn birth weight, glucose regulation, complications of pregnancy and delivery, and maternal weight retention up to 12 months postpartum.

**Methods/design:**

Fit for Delivery is a randomized controlled trial that will include 600 women expecting their first child. To be eligible, women must be 18 years of age or older, of less than 20 weeks gestational age, with a singleton pregnancy, and have a Body Mass Index (BMI) ≥ 19 kg/m^2^. The women will be randomly allocated to either an intervention group or a control group. The control group will receive standard prenatal care. The intervention group will, in addition, receive nutritional counseling by phone, access to twice-weekly exercise sessions, and information on healthy eating and physical activity provided in pamphlets, evening meetings and an interactive website. Both groups will be monitored by weighing (including bioimpedance measurements of percent body fat), blood tests, self-report questionnaires and hospital record review.

**Discussion:**

Weight gained in pregnancy affects the health of both the mother and her unborn child, and simple models for efficient intervention are in high demand. The Fit for Delivery intervention provides concrete advice on limiting energy intake and practical training in increasing physical activity. This lifestyle intervention is simple, reproducible, and inexpensive. The design of the study reflects the realities of clinical practice, where patients are free to choose whether or not they respond to health initiatives. If we find measurable health benefits associated with the intervention, it may be an easily adopted supplement to routine prenatal care, in the prevention of obesity.

**Trial registration:**

ClinicalTrial.gov, NCT01001689

## Background

The health consequences of overweight and obesity have resulted in an increased interest in maternal weight gain during pregnancy. Several authorities, including the World Health Organization, have concluded that preventive efforts among pregnant women are required to make a long-term effect on the obesity epidemic [1,2]. The American Institute of Medicine (IOM) first suggested guidelines for weight gain during pregnancy in 1990, based on a woman’s pre-pregnancy BMI [3]. Specifically, it recommended that normal weight women (BMI 19.8-26 kg/m^2^) gain 11.5-16 kg, underweight women (BMI < 19.8 kg/m^2^) gain 12.5-18 kg, overweight women (BMI 26.1-29 kg/m^2^) gain 7–11.5 kg, and obese women (BMI > 29 kg/m^2^) gain at least 6.8 kg. Research suggests that weight gain at or below these recommendations is associated with an optimal delivery outcome for both mother and child [4,5]. These guidelines were modified in 2009, and the BMI ranges now correspond with WHO definitions of normal weight (18.5-24.9 kg/m^2^), underweight (<18.5 kg/m^2^) and overweight (25–29.9 kg/m^2^), and the recommended weight gain for obese women is now 5–9 kg [6].

### The effects of excessive gestational weight gain

For the pregnant woman, excessive gestational weight gain is associated with an increased risk of complications during the prenatal period, such as gestational diabetes, gestational hypertension, and pre-eclampsia, a potentially life-threatening combination of hypertension and increased renal excretion of protein [5]. At the time of delivery, maternal overweight is associated with an increased incidence of complications, such as operative vaginal delivery, shoulder dystocia, cesarean section, and postpartum hemorrhage [7]. Excessive weight gain during pregnancy is also associated with an increased risk of weight retention after delivery [8]. In studies of obese women, as many as 73% describe pregnancy as an important trigger for a significant (>10 kilo) increase in weight [9]. Excessive gestational weight gain may also be a risk factor for the development of disease later in life, such as diabetes, hypertension, and breast cancer [10].

Excessive gestational weight gain is clearly associated with an increased incidence of large for gestational age babies [4] and has been linked with an increased incidence of overweight in childhood [11]. The large for gestational age newborn is at increased risk of birth trauma, respiratory distress syndrome, hypoglycemia, hyperbilirubinemia and admission to the neonatal intensive care unit, compared to newborns of appropriate weight [12]. Later in life, high birth weight is associated with an increased risk of overweight and obesity, along with diabetes and certain forms of cancer [13,14].

The effect of maternal weight gain on pregnancy outcome and fetal growth is at least partly moderated by maternal glucose levels. Maternal energy intake has a direct effect on serum glucose, while exercise moderates serum glucose levels by increasing skeletal muscle glucose uptake and improving insulin sensitivity [15]. Maternal plasma glucose levels have been shown to have a linear correlation with newborn birth weight [16] and the incidence of caesarean section [17]. Maternal glycemia has a direct correlation with fetal blood levels of insulin and c-peptide, which in turn regulate the growth of the fetus. Maternal dietary intake and physical activity level also have an influence on fetal levels of hormones such as leptin [18] and insulin-like growth hormone [19], the development of the fetal hypothalamus[20], the proportion of lean and fat body mass, and even gene expression [21]--all of which will affect fetal growth and energy regulation later in life.

### The current literature on interventions to limit gestational weight gain

Several recent review articles have summarized the current literature regarding interventions to limit weight gain in pregnancy [22-26]. Muktabhant et al. (2012) for the Cochrane collaboration [26] examined the results of a total of 27 randomized controlled trials, and divided interventions into those which recruited women from a general population and those that were designed for women in high risk groups. The interventions ranged from regular weighing to the use of appetite suppressants, with sample sizes of 20 to 327 participants. Only 9 studies provided some element of both diet and exercise in their intervention. Of these, five [27-31] provided behavioral counseling with recommendations for diet and exercise, one of them through an interactive computer program [30]. An additional three studies provided counseling on diet and exercise, but limited their population to overweight or obese women [32-34]. Of the 27 reported studies, only that of Hui et al. (2006) [35] provided an intervention consisting of both nutritional counseling and exercise groups. This was, however, a pilot study with only 45 participants. Hui et al. have since reported on the effects of the same intervention on a population of 190 women, and found a decreased prevalence of excessive gestational weight gain in the intervention group [36].

Postpartum weight retention was rarely reported in these interventional studies, but the Cochrane review found two studies with a combined intervention in a general population [28,29] both of which showed a significantly higher probability of returning to pre-gestational weight in the intervention arm of the study at 6 months postpartum. No studies reported on the effect of the intervention at 12 months postpartum or later. Three studies [27,29,31] reported on the effect of a combined intervention on the incidence of caesarean section, and all showed a trend toward decreased prevalence but this was not large enough to be statistically significant.

Four studies of lifestyle interventions performed in a high-risk population [29,31,33,34] and three performed on a general population [29,31,35] reported the incidence of high birth weight. Luoto et al. (2011) [33], who reported on the effect of diet and exercise counseling among women at risk for gestational diabetes, found a lower incidence of birth weight above the 90% percentile in the intervention group, but the result was not large enough to be significant in their group of 93 participants. The Cochrane review concludes that there is no evidence to date that interventions in pregnancy result in a significant reduction in excessive gestational weight gain or in the incidence of high birth weight, and calls for high quality randomized controlled trials of adequate sample size to assess potential interventions for restricting maternal weight gain [26].

Another recent meta-analysis, by Thangaratinam et al. (2012) [25], included 34 studies that examined gestational weight gain and divided interventions into three categories: diet, physical activity and mixed approach. Thangaratinam’s group found that interventions based on exercise alone showed a small but statistically significant reduction in birth weight and maternal weight gain. Interventions based on diet alone resulted in a larger decrease in maternal weight gain and a statistically significant decrease in the incidence of gestational diabetes, pre-eclampsia, gestational hypertension and preterm delivery compared with controls, but had no effect on birth weight. Mixed interventions showed a decrease in maternal weight gain compared with controls, but less than with diet alone, and no significant effect on pregnancy complications or birth weight [25]. As in the Cochrane review, the lifestyle interventions analyzed by Thangaratinam’s group consisted almost exclusively of counseling, with the exception of those reported by Hui et al. (2012) [36] and Vinter et al. (2011) [37].

Hui et al., (2012) [36] examined the effects of a lifestyle intervention with group exercise once a week and nutritional counseling in a population of 190 women in Canada, and found that fewer women in the intervention group exceeded IOM guidelines for weight gain, but found no effect on birth weight, the incidence of gestational diabetes or the frequency of caesarean section. The “Lifestyle in Pregnancy” study, published by Vinter et al. (2011) [37], included 360 obese Danish women and provided physical training once a week and dietary guidance. Vinter et al. found significantly lower gestational weight gain in the intervention group, but no difference in the incidence of gestational diabetes or caesarean section. Surprisingly, there was a significant increase in birth weight in the intervention group compared to the control group, without an increased incidence of LGA babies. Neither of these studies followed participants postpartum.

### The Fit for Delivery study

In contrast to previously published trials of interventions to limit gestational weight gain, the Fit for Delivery study combines access to twice-weekly supervised exercise sessions with counseling on nutrition and appropriate gestational weight gain, and is designed for a general population. The study is to include 600 participants, and is therefore larger than other interventions that have been published. The study is also unique in that it includes only nulliparous patients and follows all participants until 12 months after the completion of the intervention. The aim of the Fit for Delivery study is to examine if this mixed, lifestyle intervention results in a measurable decrease in maternal weight gain in pregnancy, maternal weight retention postpartum, newborn birth weight and the incidence of large for gestational age newborns, maternal hyperglycemia, and the incidence of caesarean section and operative vaginal delivery. In this article we describe the protocol for our study.

## Methods

### Study design

Fit for Delivery is a randomized controlled trial with participants allocated either to an arm which receives a lifestyle intervention in pregnancy or a control arm which receives standard care. All participants in the study will be monitored at the time of inclusion in the study, at 30 and 36 weeks of gestation, at the time of delivery and at 6 and 12 months postpartum.

### Setting

Health care during pregnancy in Norway is free of charge, and almost 100% of women receive prenatal care. Most women alternate between visits with their general practitioner and with a midwife at a local clinic. Fit for Delivery is to be conducted in the cities and towns of southern Norway, with participants from both urban and rural settings.

### Participants

All women expecting their first child and attending antenatal clinics in the included districts will be asked to participate. Midwives at the local antenatal clinics will provide information about the trial and take the initial measurement of each participant. To be eligible, women must be 18 years or older, have a BMI of 19 or higher, have a singleton pregnancy of less than 20 gestational weeks, and be fluent in either Norwegian or English. Exclusion criteria are pre-existing diabetes, physical disabilities which preclude participation in a physical fitness program (based on the recommendations of the American College of Obstetricians and Gynecologists) [38], ongoing substance abuse, and planned relocation outside the study area before delivery. Those who choose to participate will be asked to read and sign a consent form, take blood tests and answer an initial questionnaire. The participants will not receive any compensation for participation in the trial, but all examinations and interventions will be free of charge. Women who choose not to participate in the trial will be asked to answer a short questionnaire (see description below), anonymously, to learn why they are not interested in participation. Participating clinics will be asked to report each week how many women were enrolled in the trial, how many completed a non-participation questionnaire and how many declined participation without completing questionnaires.

All participants will receive two additional prenatal care visits, including ultrasound examinations, in the third trimester. Blood pressure will be examined at each visit using an Omron electronic sphygmomanometer, HEM-7301 (http://www.omron.com) following a recommended protocol for pregnant women [39].

### Intervention

The Fit for Delivery lifestyle intervention is composed of nutrition and physical activity elements.

1. Dietary counselling will consist of an initial telephone consultation with a doctor, clinical nutritionist or graduate student in public health. Counselling will be focused on ten recommendations, designed to assist in establishing good nutritional habits. Specific attention will be given to selected key behaviors: intake of fruits and vegetables, drinking water instead of drinks containing energy, regular meal patterns, and limiting consumption of snack foods and foods/drinks containing added sugar. The Fit for Delivery nutritional advice is based on recommendations from the Norwegian Directorate for Health [40], but is more specific and action-oriented. A complete discussion of the advice and its background is published elsewhere [41]. A follow-up consultation will take place 4–6 weeks after the first consultation. Participants in the intervention group will receive a pamphlet containing the 10 specific dietary recommendations. They will also have password-protected access to an interactive internet site, with information on nutrition and physical activity during pregnancy. Participants in the intervention group will be invited to two evening meetings, one with additional information about the trial and one with a hands-on cooking class, focused on the Fit for Delivery recommendations.

2. The physical activity component of the intervention is based on national [40] and international [38] guidelines and will consist of two exercise sessions each week, each lasting one hour. The groups will meet at local fitness centers, and attendance will be registered. All groups will have the same exercise plan. Exercise will be supervised either by physiotherapists or graduate students in sports science at the University of Agder. Each session will consist of 40 minutes of strength training and cardiovascular exercises of moderate intensity, measured using the Borg scale of perceived exertion [42] with 20 minutes of warm-up and stretching. Pelvic floor exercises will be performed during each session. In addition, all women in the intervention group will be encouraged to have at least one unsupervised exercise session each week, increasing to a total of 5 days each week of moderate physical activity lasting 30 minutes. Information about safe physical activity in pregnancy is provided in the pamphlet created for the trial and on the web site.

### Endpoints

The Fit for Delivery study has several endpoints.

1. Maternal weight gain and weight retention postpartum.

2. Maternal body composition at 36 weeks of gestation

3. Infant birth weight and the percent of newborns with birth weight above the 90^th^ percentile for gestational age.

4. Maternal glucose levels and measurement of hormones related to glucose metabolism.

5. Incidence of operative deliveries and delivery complications.

### Outcome measures

1. Weight and height measurements

Pre-pregnancy weight will be self-reported. Women will be weighed at their health care clinic at the time of enrollment in the study. All scales used in the project are class III with a 0.1 kg accuracy, calibrated at the initiation of the study. All participants will be weighed at Sorlandet Hospital at gestational weeks 30 and 36, wearing light indoor clothing and without shoes or socks, using a Tanita bioimpedance scale which measures weight with an accuracy of 0.1 kg and percent body fat (http://www.tanita.com/en/bc-418). The assessor will be blinded as to the woman’s group allocation. Height will be measured to the nearest centimetre (cm) at the 30 week assessment, using a Seca Leicester portable stadiometer with an accuracy of 0.1 cm. Weight at the time of delivery will be measured by labor floor staff on arrival at the labor floor using a Seca weight with a 0.1 kg graduation. Labor floor midwives will not be otherwise engaged in the study, and will be blinded as to which arm of the study her patient belongs.

The newborn will be weighed immediately following delivery on either a Seca or Solotop infant scale, each with 0.01 kg accuracy, and measured using a Seca pediatric measuring rod, with a 5 mm precision. Mother and child will be weighed at their local healthcare clinic using a class III scale, when the child has his/her 6 month and 12 month wellness examination.

2. Blood tests

Participants will be instructed to take a fasting blood sample at the office of their primary physician as soon as possible after agreeing to participate in the study, which will be analyzed at Sorlandet Hospital. Glucose will be measured, in addition to C-reactive protein, cholesterol, and triglycerides. At gestational week 30, a glucose tolerance test will be performed at Sorlandet Hospital, where serum glucose level will be measured fasting and 2 hours after intake of 75 grams of glucose. A glucose level of 7.8 mmol/l or higher at 2 hours will be defined as gestational diabetes. Positive results will be reported to the woman’s primary physician, so that the patient can be appropriately treated. Serum samples will be collected from participants at inclusion and at gestational week 30, and from the umbilical cord of babies born to women in the study, and frozen for later analysis.

3. Questionnaire

All participants in the study will complete a questionnaire at the time of inclusion in the study (approximately week 14) [see Additional file 1, toward the end of pregnancy (week 36) [see Additional file 2, 6 months post-partum [see Additional file 3 and 12 months post-partum [see Additional file 4. Women will be encouraged to answer the questionnaire electronically, with access from the Fit for Delivery web site, but a written version will also be available in both Norwegian and English. The questionnaires will include demographic variables, the short version of the International Physical Activity Questionnaire (IPAQ) [43] and a questionnaire specially designed to assess the key nutritional behaviors which are highlighted in Fit for Delivery [41]. At the time of inclusion, women will be asked to report on both current status and status before pregnancy. Subsequent questionnaires will only measure current status.

The short version of the IPAQ measures physical activity and consists of 36 questions. It has been validated in a Swedish population (both genders), where the questionnaire showed acceptable criterion validity in Swedish adults [44]. The IPAQ has been modified for the purpose of our study, in order to be answered electronically. Additional questions have been added to identify motivating factors for participation and non-participation in physical activity. The nutritional questionnaire is created for the purpose of this study and consists of 82 questions, with a food frequency section and a 24 hour recall section. The questions are designed to test selected nutritional behaviors which are emphasized during the consultation sessions. The test-retest reliability of the questionnaire was evaluated and found to be acceptable in a study including 154 pregnant women who filled out the questionnaire two weeks apart [41]. Employment information and partner’s weight will also be included. Post-partum questionnaires will contain questions about the duration and frequency of breast-feeding, as this may have an impact on post-partum weight retention.

Women who decline to participate in the study will be asked to complete a short non-response questionnaire, which consists of age, height (self-reported), pre-pregnancy weight (self-reported), smoking history, education level and reason for non-participation. This questionnaire is written and anonymous.

4. Hospital chart review

Hospital records directly related to pregnancy and delivery will be reviewed. All documented pregnancy complications will be recorded. The duration of labor and mode of delivery will be documented, as well as APGAR scores and eventual delivery complications. All admissions to the neonatal care unit will also be recorded, along with diagnosis for admission.

Women who choose to withdraw from the study will be asked if they will allow review of their hospital records, for the purpose of comparing “drop-outs” with women who remain in the study. Information regarding labor and delivery and newborn measurements will be recorded with their permission.

### Statistical analysis

A p-value of < 0.05 will be considered statistically significant. Data from each patient will be analyzed according to the principle of “intention to treat.” We will also analyze results based on the degree of attendance at exercise classes and other interventional elements. Comparisons between the endpoints and the two arms of the study will be performed using multivariate regression models; i.e. bivariate and multinominal logistic regressions, linear regression and repeated measures procedures.

### Sample size

We predicted a 20% prevalence of birth weight over 4 kg in the control group, based on 2005 statistics from the Norwegian birth registry, and claim that a reduction in prevalence of birth weight over 4 kg in the intervention group to 10% would be clinically significant. In order to demonstrate a statistical difference, we calculated that we would need 198 women in each arm of the study. Further, we expected a 10% prevalence of 2 hour glucose challenge test results ≥7.8 mmol/l in the control group, based on the findings of the Norwegian STORK study [45]. We hope to achieve a reduction to 3% prevalence of glucose ≥7.8 mmol/l in the intervention group, and maintain that this would be a clinically significant reduction. Using an alpha of 0.05 and a power of 80%, we calculated that we would need 200 women in each arm of the study in order to demonstrate a statistical difference.

We wish to examine subgroups within our participant population, specifically women with BMI >25 and women who report low levels of physical activity on their first questionnaire. We expect that our study will have a drop-out rate of approximately 25%. To compensate for these factors, we plan to recruit 300 women in each arm, in total 600 women.

### Randomization

To ensure that participants are sufficiently motivated to complete the study, they are asked to provide blood samples and answer questionnaires before enrolment, after giving informed consent. A study nurse will confirm that there is a signed consent form, blood test and completed questionnaire, and will then randomize the participant. The nurse will not check the results of either the blood test or questionnaire before randomization. A physician will check that the fasting glucose level does not indicate diabetes, which would exclude the woman from enrolment in the trial. The women will be individually randomly assigned to either the control or intervention group, based on a computer-generated list with groups of 20. Women will be randomized consecutively, based on the time of completion of all three elements needed for participation. The staff involved with providing and assessing the intervention will have no influence on the randomization procedure.

### Ethical considerations

Nutritional information and physical activity programs provided in Fit for Delivery follow national and international recommendations for safety in pregnancy. The study has been approved by the Norwegian Regional Committee for Medical Research Ethics South-East C (REK reference 2009/429). This is an independent committee, appointed by the Norwegian Ministry of Education, IRB 00001870. The study will be performed in accordance with the Helsinki Declaration, and all participation will be based on informed, written consent. At the completion of the trial, all participants in the control arm of the study will be sent the written information that was provided to the intervention arm during pregnancy.

## Discussion

There is evidence in the medical literature that excessive gestational weight gain is associated with a higher incidence of pregnancy and delivery complications, also for the woman of normal pre-pregnancy weight [5]. For women who begin pregnancy with overweight or obesity, the risks associated with excessive weight gain during gestation may be even greater [46]. Norway is a country with nationalized health care, where almost 100% of pregnant women receive prenatal care. Information about nutrition and physical activity is a part of routine pregnancy care provided by midwives. Nonetheless, there is good evidence that Norwegian women are less physically active in pregnancy than is recommended and that they gain more weight than recommended during pregnancy [47], not unlike their counterparts in Great Britain [48] and the United States [49]. The rates of overweight and obesity among young Norwegian women are also comparable to those of other developed countries. Population studies performed in the Trøndelag area of Norway (HUNT) in 2006–2008 show that 24% of women over the age of 20 are now obese, and an additional 37% are classified as overweight [50]. This is not unlike the results of the 2007–2008 National Health and Nutrition Examination Survey (NHANES) from the United States, where the corresponding figures were 35% and 29% [51].

Several authors have expressed the need for prospective, randomized studies to examine the effects of interventions to limit maternal weight gain in pregnancy [26,52]. Our hypothesis is that a lifestyle intervention in pregnancy will result in significant, measurable changes in maternal weight gain, newborn birth weight, glucose levels, and maternal weight retention the first year postpartum. We have here detailed the protocol for our randomized, controlled trial to test this hypothesis. There are several reports published to date of interventions designed to limit weight gain in pregnancy [25,26], but few studies have combined supervised group exercise sessions with clear nutritional guidelines. Thangaratinam et al. reported inferior results with mixed, lifestyle interventions compared to interventions composed of diet alone. They speculate that the inferior results of mixed interventions may be because a combined program results in less vigorous delivery of the components of the intervention, and also that it may be easier to comply with a dietary intervention alone [25]. Another element to consider is that almost all mixed interventions reported in their meta-analysis consisted of counseling alone. Other studies have found that compliance and energy expenditure are higher with group training sessions than with individual unsupervised physical activity in middle-aged women [53] and that exercise groups are associated with a greater improvement in glycemic control for diabetic patient than exercise advice [54]. A mixed intervention which includes supervised, group training may therefore be more effective in regulating weight gain and glucose levels than mixed interventions consisting of counseling alone.

The Fit for Delivery intervention combines exercise classes with nutritional counseling, and is designed for both normal-weight and overweight pregnant women. By including a general population, there is no stigma involved with participation. The intervention is also designed so that both women who have previously been sedentary and those with an active lifestyle will be able to perform the exercises and follow the nutritional advice, albeit at different levels. By recruiting all first-time mothers at local health care clinics, we hope to include a broad and diverse population that will reflect a standard population of pregnant women. Results are primarily to be analyzed by the principle of “intention to treat”, so that we will measure the effect of providing this extra treatment in pregnancy, with varying compliance. This reflects the realities of health care, where patients are free to decide if they wish to partake of the treatments which are available to them. We have emphasized a simple and inexpensive design for the Fit for Delivery intervention that can easily be adopted by health authorities should it be proven effective.

Pregnancy is a unique opportunity to affect the health of both mother and child. Other authors have described pregnancy as a “teachable moment”: women have a new awareness of their body and the responsibility of a new life, and are therefore more responsive to healthcare information than at other times [55]. The WHO has listed both pregnancy and the prenatal period as key moments for lifetime risk of obesity [2]. By preventing excessive weight gain during pregnancy, there will be less risk of the mother developing obesity later in life. Perhaps more important, a lifestyle change during pregnancy has the potential to affect the health of the newborn at the earliest possible stage—while still in the uterus. By teaching women new habits, the intervention will also influence the environment the child will enter after birth. Should our Fit for Delivery intervention demonstrate measurable health benefits for mother and child, it may be of use in the important goal of curbing the obesity epidemic.

## Competing interest

The authors declare that they have no competing interests. The authors have no conflict of interest with the fitness centers which will provide training facilities for the study.

## Authors’ contributions

LRS, NØ, EB, HLS, TH and IV designed the study. HLS and LRS have developed the physical activity information for Fit for Delivery, MT and HLS supervise the physical activity portion of the intervention, while NØ and EB have developed the nutritional counseling and nutritional questionnaires included in Fit for Delivery study. LRS will manage the obstetrical care (examination and ultrasound) of participants, supervised by IV and TH. All authors read and approved the final manuscript.

## Pre-publication history

The pre-publication history for this paper can be accessed here:

http://www.biomedcentral.com/1471-2458/13/132/prepub

## Supplementary Material

Additional file 1 The written, English version of the questionnaire which is completed at inclusion in the study. Click here for file

Additional file 2 The written, English version of the questionnaire which is completed at approximately gestational week 36. Click here for file

Additional file 3 The written, English version of the questionnaire which is completed at 6 months postpartum. Click here for file

Additional file 4 The written, English version of the questionnaire which is completed at 6 months postpartum. Click here for file

## References

[B1] Wojcicki JM Heyman MB Let’s Move–childhood obesity prevention from pregnancy and infancy onward N Engl J Med 2010 362 16 1457 1459 20393165 10.1056/NEJMp1001857 PMC3250598

[B2] World Health Organization Obesity: Preventing and Managing the Global Epidemic 2000 Geneva: World Health Organization 11234459

[B3] Institute of Medicine Nutrition During Pregnancy 1990 Washington DC: National Academies Press

[B4] DeVader SR Neeley HL Myles TD Leet TL Evaluation of gestational weight gain guidelines for women with normal prepregnancy body mass index Obstet Gynecol 2007 110 4 745 751 17906004 10.1097/01.AOG.0000284451.37882.85

[B5] Thorsdottir I Torfadottir JE Birgisdottir BE Geirsson RT Weight gain in women of normal weight before pregnancy: complications in pregnancy or delivery and birth outcome Obstet Gynecol 2002 99 5 Pt 1 799 806 11978290 10.1016/s0029-7844(02)01946-4

[B6] Institute of Medicine and National Research Committee to Reexamine IOM Pregnancy Weight Guidelines Weight Gain During Pregnancy: Reexamining the Guidelines 2009 Washington, DC: National Academies Press

[B7] Andreasen KR Andersen ML Schantz AL Obesity and pregnancy Acta Obstet Gynecol Scand 2004 83 11 1022 1029 15488115 10.1111/j.0001-6349.2004.00624.x

[B8] Linne Y Dye L Barkeling B Rossner S Long-term weight development in women: a 15-year follow-up of the effects of pregnancy Obes Res 2004 12 7 1166 1178 15292482 10.1038/oby.2004.146

[B9] Linne Y Rossner S Interrelationships between weight development and weight retention in subsequent pregnancies: the SPAWN study Acta Obstet Gynecol Scand 2003 82 4 318 325 12716315 10.1080/j.1600-0412.2003.00150.x

[B10] Kinnunen TI Pasanen M Aittasalo M Fogelholm M Hilakivi-Clarke L Weiderpass E Luoto R Preventing excessive weight gain during pregnancy - a controlled trial in primary health care Eur J Clin Nutr 2007 61 7 884 891 17228348 10.1038/sj.ejcn.1602602

[B11] Wrotniak BH Shults J Butts S Stettler N Gestational weight gain and risk of overweight in the offspring at age 7 y in a multicenter, multiethnic cohort study Am J Clin Nutr 2008 87 6 1818 1824 18541573 10.1093/ajcn/87.6.1818

[B12] Henriksen T The macrosomic fetus: a challenge in current obstetrics Acta Obstet Gynecol Scand 2008 87 2 134 145 18231880 10.1080/00016340801899289

[B13] Moschonis G Grammatikaki E Manios Y Perinatal predictors of overweight at infancy and preschool childhood: the GENESIS study Int J Obes (Lond) 2008 32 1 39 47 18059407 10.1038/sj.ijo.0803764

[B14] Henriksen T Nutrition, weight and pregnancy TidsskrNor Laegeforen 2007 127 18 2399 2401 17895947

[B15] Hawley JA Lessard SJ Exercise training-induced improvements in insulin action Acta Physiol (Oxf) 2008 192 1 127 135 18171435 10.1111/j.1748-1716.2007.01783.x

[B16] Hyperglycemia and Adverse Pregnancy Outcome (HAPO) Study Associations with neonatal anthropometrics Diabetes 2009 58 2 453 459 19011170 10.2337/db08-1112 PMC2628620

[B17] Catalano PM McIntyre HD Cruickshank JK McCance DR Dyer AR Metzger BE Lowe LP Trimble ER Coustan DR Hadden DR The hyperglycemia and adverse pregnancy outcome study: associations of GDM and obesity with pregnancy outcomes Diabetes Care 2012 35 4 780 786 22357187 10.2337/dc11-1790 PMC3308300

[B18] Jansson N Nilsfelt A Gellerstedt M Wennergren M Rossander-Hulthen L Powell TL Jansson T Maternal hormones linking maternal body mass index and dietary intake to birth weight Am J Clin Nutr 2008 87 6 1743 1749 18541564 10.1093/ajcn/87.6.1743

[B19] Hopkins SA Baldi JC Cutfield WS McCowan L Hofman PL Exercise training in pregnancy reduces offspring size without changes in maternal insulin sensitivity J Clin Endocrinol Metab 2010 95 5 2080 2088 20335449 10.1210/jc.2009-2255

[B20] Sullivan EL Smith MS Grove KL Perinatal exposure to high-fat diet programs energy balance, metabolism and behavior in adulthood Neuroendocrinology 2011 93 1 1 8 21079387 10.1159/000322038 PMC3700139

[B21] Alfaradhi MZ Ozanne SE Developmental programming in response to maternal overnutrition Front Genet 2011 2 27 22303323 10.3389/fgene.2011.00027 PMC3268582

[B22] Tanentsapf I Heitmann BL Adegboye AR Systematic review of clinical trials on dietary interventions to prevent excessive weight gain during pregnancy among normal weight, overweight and obese women BMC Pregnancy Childbirth 2011 11 1 81 22029725 10.1186/1471-2393-11-81 PMC3215955

[B23] Ronnberg A Nilsson K Interventions during pregnancy to reduce excessive gestational weight gain: a systematic review assessing current clinical evidence using the Grading of Recommendations, Assessment, Development and Evaluation (GRADE) system BJOG 2010 117 11 1327 1334 20840691 10.1111/j.1471-0528.2010.02619.x

[B24] Skouteris H Hartley-Clark L McCabe M Milgrom J Kent B Herring SJ Gale J Preventing excessive gestational weight gain: a systematic review of interventions Obes Rev 2010 11 11 757 768 20880128 10.1111/j.1467-789X.2010.00806.x

[B25] Thangaratinam S Rogozinska E Jolly K Glinkowski S Roseboom T Tomlinson JW Kunz R Mol BW Coomarasamy A Khan KS Effects of interventions in pregnancy on maternal weight and obstetric outcomes: meta-analysis of randomised evidence BMJ 2012 344 e2088 22596383 10.1136/bmj.e2088 PMC3355191

[B26] Muktabhant B Lumbiganon P Ngamjarus C Dowswell T Interventions for preventing excessive weight gain during pregnancy Cochrane Database Syst Rev 2012 4 CD007145 22513947 10.1002/14651858.CD007145.pub2 PMC4163963

[B27] Asbee SM Jenkins TR Butler JR White J Elliot M Rutledge A Preventing excessive weight gain during pregnancy through dietary and lifestyle counseling: a randomized controlled trial Obstet Gynecol 2009 113 2 Pt 1 305 312 19155899 10.1097/AOG.0b013e318195baef

[B28] Huang TT Yeh CY Tsai YC A diet and physical activity intervention for preventing weight retention among Taiwanese childbearing women: a randomised controlled trial Midwifery 2011 27 2 257 264 19775782 10.1016/j.midw.2009.06.009

[B29] Phelan S Phipps MG Abrams B Darroch F Schaffner A Wing RR Randomized trial of a behavioral intervention to prevent excessive gestational weight gain: the Fit for Delivery Study Am J Clin Nutr 2011 93 4 772 779 21310836 10.3945/ajcn.110.005306 PMC3057546

[B30] Jackson RA Stotland NE Caughey AB Gerbert B Improving diet and exercise in pregnancy with Video Doctor counseling: a randomized trial Patient Educ Couns 2011 83 2 203 209 21459255 10.1016/j.pec.2010.05.019

[B31] Polley BA Wing RR Sims CJ Randomized controlled trial to prevent excessive weight gain in pregnant women Int J Obes Relat Metab Disord 2002 26 11 1494 1502 12439652 10.1038/sj.ijo.0802130

[B32] Korpi-Hyovalti EA Laaksonen DE Schwab US Vanhapiha TH Vihla KR Heinonen ST Niskanen LK Feasibility of a lifestyle intervention in early pregnancy to prevent deterioration of glucose tolerance BMC Publ Health 2011 11 179 10.1186/1471-2458-11-179 PMC3078095 21429234

[B33] Luoto R Kinnunen TI Aittasalo M Kolu P Raitanen J Ojala K Mansikkamaki K Lamberg S Vasankari T Komulainen T Primary prevention of gestational diabetes mellitus and large-for-gestational-age newborns by lifestyle counseling: a cluster-randomized controlled trial PLoS Med 2011 8 5 e1001036 21610860 10.1371/journal.pmed.1001036 PMC3096610

[B34] Guelinckx I Devlieger R Mullie P Vansant G Effect of lifestyle intervention on dietary habits, physical activity, and gestational weight gain in obese pregnant women: a randomized controlled trial Am J Clin Nutr 2010 91 2 373 380 19955397 10.3945/ajcn.2009.28166

[B35] Hui ALS Gardiner P Sevenhuysen G Murray R Morris M Report of a pilot study on community-based exercise and dietary intervention during pregnancy Canadian Journal of Diabetes 2006 30 169 175

[B36] Hui A Back L Ludwig S Gardiner P Sevenhuysen G Dean H Sellers E McGavock J Morris M Bruce S Lifestyle intervention on diet and exercise reduced excessive gestational weight gain in pregnant women under a randomised controlled trial BJOG 2012 119 1 70 77 22017967 10.1111/j.1471-0528.2011.03184.x

[B37] Vinter CA Jensen DM Ovesen P Beck-Nielsen H Jorgensen JS The LiP (Lifestyle in Pregnancy) study: a randomized controlled trial of lifestyle intervention in 360 obese pregnant women Diabetes Care 2011 34 12 2502 2507 21972411 10.2337/dc11-1150 PMC3220844

[B38] ACOG Committee opinion Number 267, January 2002: Exercise during pregnancy and the postpartum period Obstet Gynecol 2002 99 1 171 173 11777528 10.1016/s0029-7844(01)01749-5

[B39] Higgins JR de Swiet M Blood-pressure measurement and classification in pregnancy Lancet 2001 357 9250 131 135 11197413 10.1016/S0140-6736(00)03552-2

[B40] The Norwegian Directorate of Health Pregnant 2012 Oslo

[B41] Overby NC Hillesund ER Sagedal LR Vistad I Bere E The Fit for Delivery Study: rationale for the recommendations and test-retest reliability of a dietary score measuring adherence to 10 specific recommendations for prevention of excessive weight gain during pregnancy Matern Child Nutr 2012 10.1111/mcn.12026, epub ahead of print 10.1111/mcn.12026 PMC6860359 23241065

[B42] Borg E Kaijser L A comparison between three rating scales for perceived exertion and two different work tests Scand J Med Sci Sports 2006 16 1 57 69 16430682 10.1111/j.1600-0838.2005.00448.x

[B43] Craig CL Marshall AL Sjostrom M Bauman AE Booth ML Ainsworth BE Pratt M Ekelund U Yngve A Sallis JF International physical activity questionnaire: 12-country reliability and validity Med Sci Sports Exerc 2003 35 8 1381 1395 12900694 10.1249/01.MSS.0000078924.61453.FB

[B44] Ekelund U Sepp H Brage S Becker W Jakes R Hennings M Wareham NJ Criterion-related validity of the last 7-day, short form of the International Physical Activity Questionnaire in Swedish adults Public Health Nutr 2006 9 2 258 265 16571181 10.1079/phn2005840

[B45] Jenum AK Sletner L Voldner N Vangen S Morkrid K Andersen LF Nakstad B Skrivarhaug T Rognerud-Jensen OH Roald B The STORK Groruddalen research programme: a population-based cohort study of gestational diabetes, physical activity, and obesity in pregnancy in a multiethnic population. Rationale, methods, study population, and participation rates Scand J Public Health 2010 38 5 Suppl 60 70 21062840 10.1177/1403494810378921

[B46] Cedergren M Effects of gestational weight gain and body mass index on obstetric outcome in Sweden Int J Gynaecol Obstet 2006 93 3 269 274 16626716 10.1016/j.ijgo.2006.03.002

[B47] Haakstad LA Voldner N Henriksen T Bo K Physical activity level and weight gain in a cohort of pregnant Norwegian women Acta Obstet Gynecol Scand 2007 86 5 559 564 17464584 10.1080/00016340601185301

[B48] Guelinckx I Devlieger R Beckers K Vansant G Maternal obesity: pregnancy complications, gestational weight gain and nutrition Obes Rev 2008 9 2 140 150 18221480 10.1111/j.1467-789X.2007.00464.x

[B49] Chu SY Callaghan WM Bish CL D’Angelo D Gestational weight gain by body mass index among US women delivering live births, 2004–2005: fueling future obesity Am J Obstet Gynecol 2009 200 3 271 e271 277 19136091 10.1016/j.ajog.2008.09.879

[B50] Krokstad S, Knudtsen MS Public Health Development. The HUNT Study, Norway. HUNT 1 (1984-86) - HUNT 2 (1995-97) - HUNT 3 (2006-08) 2011 Levanger: HUNT Research Center, NTNU

[B51] Flegal KM Carroll MD Ogden CL Curtin LR Prevalence and trends in obesity among US adults, 1999–2008 JAMA 2010 303 3 235 241 20071471 10.1001/jama.2009.2014

[B52] Phelan S Jankovitz K Hagobian T Abrams B Reducing excessive gestational weight gain: lessons from the weight control literature and avenues for future research Womens Health (Lond Engl) 2011 7 6 641 661 22040207 10.2217/whe.11.70

[B53] Cox KL Burke V Gorely TJ Beilin LJ Puddey IB Controlled comparison of retention and adherence in home- vs center-initiated exercise interventions in women ages 40–65 years: the S.W.E.A.T. Study (Sedentary Women Exercise Adherence Trial) Prev Med 2003 36 1 17 29 12473421 10.1006/pmed.2002.1134

[B54] Umpierre D Ribeiro PA Kramer CK Leitao CB Zucatti AT Azevedo MJ Gross JL Ribeiro JP Schaan BD Physical activity advice only or structured exercise training and association with HbA1c levels in type 2 diabetes: a systematic review and meta-analysis JAMA 2011 305 17 1790 1799 21540423 10.1001/jama.2011.576

[B55] Phelan S Pregnancy: a “teachable moment” for weight control and obesity prevention Am J Obstet Gynecol 2010 202 2 135 138 19683692 10.1016/j.ajog.2009.06.008 PMC2815033

