# Supplementary material for: Study protocol: fit for delivery - can a lifestyle intervention in pregnancy result in measurable health benefits for mothers and newborns? A randomized controlled trial
Source: BMC Public Health. 2013 Feb 13;13:132. doi: 10.1186/1471-2458-13-132 (PMC3577450; doi:10.1186/1471-2458-13-132)
Supplement: Additional file 1 — The written, English version of the questionnaire which is completed at inclusion in the study. [file 1471-2458-13-132-S1.docx]

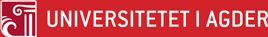

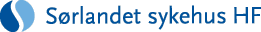


Participant number:

Fit for Delivery

Thank you for choosing to participate in our research study “Fit for Delivery”!
We look forward to following you through your pregnancy and first year as a new mother.

Please take about 30 minutes of your time and complete this survey. Read the questions carefully, and answer as best you can. Use black or blue ink, and make an “X” inside the box. Write clearly, where necessary.

The survey can be delivered in an envelope to the midwife or secretary at your healthcare clinic, or mailed to us directly (stamped, addressed envelopes are available at the healthcare clinic). In all cases, your answers will be treated confidentially—your answers can not be traced back to you.

**Thank you for your help!**

Sincerely,

*The Fit for Delivery* Team


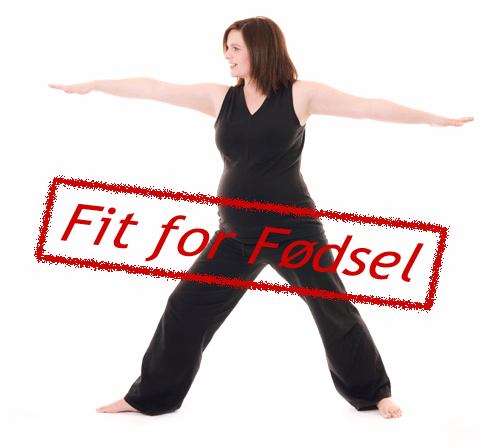


Date of completion:­­­­­­­­­­­­­__________________

FFF-start

Some questions about you: (ver. 090112)

### 001. Participant number (please write clearly):

__________________________

### 002. What is your date of birth?

__________________________

### 003. How far along are you in your pregnancy?

_______________ weeks

### 004. What was your weight immediately before becoming pregnant?

_______ kg

### 005. How tall are you? (in cm)

_______cm

### 006. With whom do you live?

husband/partner/
boyfriend

parents

friends

live alone

### 007. If you live with the baby’s father, how tall is he? (in cm)

______________ cm

### 008. If you live with the baby’s father, what is his weight?

______________ kg

### 009. What is your highest level of education? (Choose one)

Less than 7 years of primary education

Primary education, 7-10 years

Trade school or 1-2 years

of secondary education

3 years of secondary education

College/university,

Less than 4 years

College/university,

4 years or more

### 010. What is your primary activity?

Working outside the home

Student

Unemployed

Prolonged sick leave/disabled

Homemaker

### 011. What was your household’s combined income last year? Include all income from wages, disability payments, social assistance, investment dividends, etc. (in krone)

- Under 125 000
- 125 000-200 000
- 201 000-300 000
- 301 000-400 000
- 401 000-550 000
- 551 000-700 000
- 701 000 -850 000
- Over 850 000
- Do not wish to answer

**012. Have you been on a diet within the last year?**

No, my weight is fine

No, but I needed to lose weight

Yes

**013. Do you smoke?**

Never smoked

Smoked before I became pregnant, but have stopped completely

Smoke 1-4 cigs / day

Smoke 5-9 cigs / day

Smoke 10-20 cigs / day

Smoke > 20 cigs / day

**014. Do you use snuff?**

Have never used snuff

Used snuff occasionally before I became pregnant, but have stopped completely

Used snuff regularly before I became pregnant, but have stopped completely

Use snuff occasionally

Use snuff daily, about

**015**. _____ doses per day

**016. Do you use any medication daily?**

No

Yes

**017. If yes, which? (Name of medication):**

**018. Do you use any vitamins or supplements daily?**

No

Yes

**019. If yes, which? (name of supplement- iron, folate, etc.):**

**020. Have you ever used any form of drugs/narcotics?**

Never tried

Used drugs regularly before I became pregnant, but have stopped completely

Have tried drugs in the past, but have stopped completely

Use drugs occasionally

Use drugs on a weekly basis

**021. If yes, which? (name of drug/ narcotic):**

### 022. How would you describe your own health? (choose one):

Very good

Good

Neither good nor bad

Poor

Very poor

**023. To what extent does your health limit your activities of daily life? (choose one):**

To a large extent

To some extent

Very little

Not at all

**024. If you are employed outside the home, have you had more than 1 week of sick leave during the past month ?**

Yes

No

**025. If yes, how long have you had sick leave?** (choose the answer that fits best):

1-2 weeks, partial sick leave

1-2 weeks, complete sick leave

2-3 weeks, partial sick leave

2-3 weeks, complete sick leave

3-4 weeks, partial sick leave

3-4 weeks, complete sick leave

4+ weeks, partial sick leave

4+ weeks, complete sick leave

Physical activity

We would now like to ask you about the physical activities you do. We are interested in information about different kinds of physical activity that are part of women’s daily lives. Please answer all questions, regardless of how active you believe yourself to be. Think of activities you do at work, as part of your house and yard work, to get from place to place, and in your spare time (for recreation, exercise or sport). We ask two sets of questions: We would first like you to tell us about your activities during **the last seven days**. After that, we are interested in your physical situation in **the period right before you became pregnant**.

Think of all **vigorous** physical activities you have performed over the last 7 days. Vigorous physical activities refer to activities that take hard physical effort and make you breathe much harder than normal. Include only those activities that last for at least 10 minutes at a time.

**026. During the last 7 days, on how many days did you do vigorous physical activities like heavy lifting, digging, aerobics or fast biking?**

_____ days

No vigorous physical activities: *Go to question 28.*

**027. How much time did you usually spend on one of those days doing vigorous physical activities?**

0. Don’t know

1. 10 minutes

2. 20 minutes

3. 30 minutes

4. 40 minutes

5. 50 minutes

6. 1 hour

7. 1 hour and 10 minutes

8. 1 hour and 20 minutes

9. 1 hour and 30 minutes

10. 1 hour and 40 minutes

11. 1 hour and 50 minutes

12. 2 hours or more

Think of all **moderate** physical activities you have done over the last 7 days. Moderate physical activities are activities that take moderate physical effort and make you breathe somewhat harder than normal. Include only those activities that last for at least 10 minutes at a time.

**028. During the last 7 days, on how many days did you do moderate physical activities like carrying light loads, bicycling at a regular pace, or light jogging? Do not include walking.**

_____ days

No moderate physical activities: *Go to question 30.*

**029. How much time did you usually spend on one of those days doing moderate physical activities?**

0. Don’t know

1. 10 minutes

2. 20 minutes

3. 30 minutes

4. 40 minutes

5. 50 minutes

6. 1 hour

7. 1 hour and 10 minutes

8. 1 hour and 20 minutes

9. 1 hour and 30 minutes

10. 1 hour and 40 minutes

11. 1 hour and 50 minutes

12. 2 hours or more

Think about the time you have spent walking during the last 7 days. This includes walking at work and at home, walking to travel from place to place, and any other walking that you did solely for recreation, sport, exercise or leisure.

**030.** During the last 7 days, on how many days did you walk for at least 10 minutes at a time?

_____ days

Didn’t walk: *Go to question 32.*

**031. How much time in total did you usually spend walking on one of those days?**

0. Don’t know

1. 10 minutes

2. 20 minutes

3. 30 minutes

4. 40 minutes

5. 50 minutes

6. 1 hour

7. 1 hour and 10 minutes

8. 1 hour and 20 minutes

9. 1 hour and 30 minutes

10. 1 hour and 40 minutes

11. 1 hour and 50 minutes

12. 2 hours or more

The next question is about the time you spent sitting on weekdays while at work, at home, while doing course work and during leisure time. This includes time spent sitting at a desk, visiting friends, reading, traveling on a bus or sitting or lying down to watch television.

**032. During the last 7 days, how much time in total did you usually spend sitting on a week day?**

Answer: _______ hours

We would now like you to think back to the time right before you became pregnant. Try to imagine a typical week during that time. Think of activities you did at work, as part of your house and yard work, to get from place to place, and in your spare time (for recreation, exercise or sport).

Think of all **vigorous** physical activities you performed during a typical week (7 days) right before you became pregnant. Vigorous physical activities refer to activities that take hard physical effort and make you breathe much harder than normal. Include only those activities that last for at least 10 minutes at a time.

**033. How many days during a typical week did you do vigorous physical activities like heavy lifting, digging, aerobics or fast biking?**

_____ days

No vigorous physical activities: *Go to question 35.*

**034. How much time did you usually spend on one of those days doing vigorous physical activities?**

0. Don’t know

1. 10 minutes

2. 20 minutes

3. 30 minutes

4. 40 minutes

5. 50 minutes

6. 1 hour

7. 1 hour and 10 minutes

8. 1 hour and 20 minutes

9. 1 hour and 30 minutes

10. 1 hour and 40 minutes

11. 1 hour and 50 minutes

12. 2 hours or more

Think of all **moderate** physical activities you performed during a typical week (7 days) **right before you became pregnant**. Moderate physical activities are activities that take moderate physical effort and make you breathe somewhat harder than normal. Include only those activities that last for at least 10 minutes at a time.

**035. How many days during a typical week did you do moderate physical activities like carrying light loads, bicycling at a regular pace, or light jogging? Do not include walking.**

_____ days

No moderate physical activities: *Go to question 37.*

**036. How much time did you usually spend on one of those days doing moderate physical activities?**

0. Don’t know

1. 10 minutes

2. 20 minutes

3. 30 minutes

4. 40 minutes

5. 50 minutes

6. 1 hour

7. 1 hour and 10 minutes

8. 1 hour and 20 minutes

9. 1 hour and 30 minutes

10. 1 hour and 40 minutes

11. 1 hour and 50 minutes

12. 2 hours or more

Think about the time you have spent **walking** during a typical week (7 days) **right before you became pregnant**. This includes walking at work and at home, walking to travel from place to place, and any other walking that you did solely for recreation, sport, exercise or leisure.

**037. How many days during a typical week did you walk for at least 10 minutes at a time?**

_____ days

Didn’t walk: *Go to question 39.*

**038. How much time in total did you usually spend walking on one of those days?**

0. Don’t know

1. 10 minutes

2. 20 minutes

3. 30 minutes

4. 40 minutes

5. 50 minutes

6. 1 hour

7. 1 hour and 10 minutes

8. 1 hour and 20 minutes

9. 1 hour and 30 minutes

10. 1 hour and 40 minutes

11. 1 hour and 50 minutes

12. 2 hours or more

The next question is about the time you spent **sitting** on weekdays during a typical week (7 days) before you became pregnant. Include time you spent sitting while at work, at home, while doing course work and during leisure time. This includes time spent sitting at a desk, visiting friends, reading, traveling on a bus or sitting or lying down to watch television.

**039. How much time in total did you usually spend sitting on a week day during a typical week?**

Answer: _______ hours

**(040-042) Think further back in time. How often did you do a sport or physical activity that was so intense that you became sweaty and/or breathless when you were: *(Make a mark for each age group)?***

**When I was When I was When I was between**

**younger than 10: 10 to 14: 15 and 20:**

Never

Less than 1 x/month

1-3 x/month

1 /week

2-3 x/week

4-6 x/week

Every day

### How do you usually get to work/school?

**Before pregnancy: Now:**

**(043): (044):**

Walk

Bike

Public transportation (bus, train, etc.)

Car

Motorcycle, scooter or moped

Not applicable (not working, going to school)

**Below you will find a list of reasons for NOT doing physical activities.**

Please mark one or more boxes for the reason(s) that are most important for you:

**Before pregnancy: Now:**

**(045/065)** Don’t have the time

**(046/066)** Can’t afford it

**(047/067)** Transportation problems

**(048/068)** Negative experiences

**(049/069)** Problems with mobility

**(050/070)** Don’t think I can do it

**(051/071)** Don’t have the energy

**(052/072)** Afraid to get hurt (to fall, get a sprain)

**(053/073)** Would rather use my time on other things

**(054/074)** Because of my physical health

**(055/075)** Don’t have anyone to do physical activities with me

**(056/076)** Schedules don’t fit for me

**(057/077)** Don’t know of anything available to me

**(058/078)** Afraid to go out

**(059/079)** Nothing available in my area of interest

**(060/080)** Because of nausea

**(061/081)** Fear of urinary incontinence

**(062/082)** Afraid to harm the baby

**(063/083)** Pelvic pain

If you have other reasons, please explain:

**064.** Before pregnancy: _______________________________________________________

**085.** Now: _______________________________________________________

What do you usually eat?

When you answer these questions, think about what you usually ate and drank before you became pregnant and what you eat and drink now. Consider what you eat at home, at work, and in your spare time. Mark the box that you feel best fits for you. Please answer every question for both before pregnancy and now.

### (086-087). How often did you/do you eat breakfast?

**Before Now**

- Never
- Less than once a week

Once a week

Twice a week

3 times a week

4 times a week

5 times a week

6 times a week

Every day

**(088-089). How often did you/do you eat lunch?**

**Before Now**

- Never
- Less than once a week

Once a week

Twice a week

3 times a week

4 times a week

5 times a week

6 times a week

Every day

### (090-091). How often did you/do you eat dinner?

**Before Now**

- Never
- Less than once a week

Once a week

Twice a week

3 times a week

4 times a week

5 times a week

6 times a week

Every day

### (092-093). How often did you/do you eat a late supper (kveldsmat)?

**Before Now**

- Never
- Less than once a week

Once a week

Twice a week

3 times a week

4 times a week

5 times a week

6 times a week

Every day

### (094-095). How often did you/do you eat snacks?

**Before Now**

- Never
- Less than once a week

Once a week

Twice a week

3 times a week

4 times a week

5 times a week

6 times a week

Every day

Several times each
day

### (096-097). How often did you/do you drink whole milk?

**Before Now**

- Never
- Less than once a week

Once a week

Twice a week

3 times a week

4 times a week

5 times a week

6 times a week

Every day

Several times each
day

### (098-099). How often did you/do you drink low-fat milk?

**Before Now**

- Never
- Less than once a week

Once a week

Twice a week

3 times a week

4 times a week

5 times a week

6 times a week

Every day

Several times each
day

### (100-101). How often did you/do you drink skimmed milk?

**Before Now**

- Never
- Less than once a week

Once a week

Twice a week

3 times a week

4 times a week

5 times a week

6 times a week

Every day

Several times each
day

### (102-103). How often did you/do you drink juice?

**Before Now**

- Never
- Less than once a week

Once a week

Twice a week

3 times a week

4 times a week

5 times a week

6 times a week

Every day

Several times each
day

### (104-105). How often did you/do you drink fruit nectar?

**Before Now**

- Never
- Less than once a week

Once a week

Twice a week

3 times a week

4 times a week

5 times a week

6 times a week

Every day

Several times each
day

### (106-107). How often did you/do you drink soda/punch – *with sugar*?

**Before Now**

- Never
- Less than once a week

Once a week

Twice a week

3 times a week

4 times a week

5 times a week

6 times a week

Every day

Several times each
day

### (108-109). How often did you/do you drink soda/punch—*without sugar*?

**Before Now**

- Never
- Less than once a week

Once a week

Twice a week

3 times a week

4 times a week

5 times a week

6 times a week

Every day

Several times each
day

### (110-111). How often did you/do you drink beverages that contain alcohol?

**Before Now**

- Never
- Less than once a week

Once a week

Twice a week

3 times a week

4 times a week

5 times a week

6 times a week

Every day

Several times each
day

### (112-113). How often did you/do you drink tap water?

**Before Now**

- Never
- Less than once a week

Once a week

Twice a week

3 times a week

4 times a week

5 times a week

6 times a week

Every day

Several times each
day

### (114-115). How often did you/do you drink bottled water (without carbonation or flavor added)?

**Before Now**

- Never
- Less than once a week

Once a week

Twice a week

3 times a week

4 times a week

5 times a week

6 times a week

Every day

Several times each
day

### (116-117). How often did you/do you drink bottled water with carbonation or flavor added?

**Before Now**

- Never
- Less than once a week

Once a week

Twice a week

3 times a week

4 times a week

5 times a week

6 times a week

Every day

Several times each
day

### (118-119). How often did you/do you drink coffee?

**Before Now**

- Never
- Less than once a week

Once a week

Twice a week

3 times a week

4 times a week

5 times a week

6 times a week

Every day

Several times each
day

### (120-121). How often did you/do you eat potatoes?

**Before Now**

- Never
- Less than once a week

Once a week

Twice a week

3 times a week

4 times a week

5 times a week

6 times a week

Every day

Several times each
day

### (122-123). How often did you/do you eat vegetables at dinner?

**Before Now**

- Never
- Less than once a week

Once a week

Twice a week

3 times a week

4 times a week

5 times a week

6 times a week

Every day

Several times each
day

### (124-125). How often did you/do you eat vegetables on your sandwich?

**Before Now**

- Never
- Less than once a week

Once a week

Twice a week

3 times a week

4 times a week

5 times a week

6 times a week

Every day

Several times each
day

### (126-127). How often did you/do you eat other vegetables (for example, carrots at lunchtime)?

**Before Now**

- Never
- Less than once a week

Once a week

Twice a week

3 times a week

4 times a week

5 times a week

6 times a week

Every day

Several times each
day

### (128-129). How often did you/do you eat apples, oranges, pears or bananas?

**Before Now**

- Never
- Less than once a week

Once a week

Twice a week

3 times a week

4 times a week

5 times a week

6 times a week

Every day

Several times each
day

### (130-131). How often did you/do you eat other fruits or berries (fruits or berries other than apples, oranges, pears or bananas)?

**Before Now**

- Never
- Less than once a week

Once a week

Twice a week

3 times a week

4 times a week

5 times a week

6 times a week

Every day

Several times each
day

### (132-133). How often did you/do you eat fruits or vegetables as snacks?

**Before Now**

- Never
- Less than once a week

Once a week

Twice a week

3 times a week

4 times a week

5 times a week

6 times a week

Every day

Several times each
day

### (134-135). How often did you/do you eat cookies or crackers?

**Before Now**

- Never
- Less than once a week

Once a week

Twice a week

3 times a week

4 times a week

5 times a week

6 times a week

Every day

Several times each
day

### (136-137). How often did you/do you eat sweet buns (sweet rolls, “boller”, etc)?

**Before Now**

- Never
- Less than once a week

Once a week

Twice a week

3 times a week

4 times a week

5 times a week

6 times a week

Every day

Several times each
day

### (138-139). How often did you/do you eat cake, muffins, etc.?

**Before Now**

- Never
- Less than once a week

Once a week

Twice a week

3 times a week

4 times a week

5 times a week

6 times a week

Every day

Several times each
day

### (140-141). How often did you/do you eat cereal without added sugar?

**Before Now**

- Never
- Less than once a week

Once a week

Twice a week

3 times a week

4 times a week

5 times a week

6 times a week

Every day

Several times each
day

### (142-143). How often did you/do you eat cereal containing sugar?

**Before Now**

- Never
- Less than once a week

Once a week

Twice a week

3 times a week

4 times a week

5 times a week

6 times a week

Every day

Several times each
day

### (144-145). How often did you/do you eat plain yogurt (yogurt without added sugar)?

**Before Now**

- Never
- Less than once a week

Once a week

Twice a week

3 times a week

4 times a week

5 times a week

6 times a week

Every day

Several times each
day

### (146-147). How often did you/do you eat yogurt with added sugar?

**Before Now**

- Never
- Less than once a week

Once a week

Twice a week

3 times a week

4 times a week

5 times a week

6 times a week

Every day

Several times each
day

### (148-149). How often did you/do you eat instant noodles (for example, Mr. Lee)?

**Before Now**

- Never
- Less than once a week

Once a week

Twice a week

3 times a week

4 times a week

5 times a week

6 times a week

Every day

Several times each
day

### (150-151). How often did you/do you eat potato chips/other salty snacks?

**Before Now**

- Never
- Less than once a week

Once a week

Twice a week

3 times a week

4 times a week

5 times a week

6 times a week

Every day

Several times each
day

### (152-153). How often did you/do you eat chocolate/ other sweets?

**Before Now**

- Never
- Less than once a week

Once a week

Twice a week

3 times a week

4 times a week

5 times a week

6 times a week

Every day

Several times each
day

### (154-155). How often did you/do you eat hot dogs/ sausages from a gas station or kiosk?

**Before Now**

- Never
- Less than once a week

Once a week

Twice a week

3 times a week

4 times a week

5 times a week

6 times a week

Every day

Several times each
day

### (156-157). How often did you/do you eat french fries from a fast-food chain?

**Before Now**

- Never
- Less than once a week

Once a week

Twice a week

3 times a week

4 times a week

5 times a week

6 times a week

Every day

Several times each
day

### (158-159). How often did you/do you add sugar to the food you eat?

**Before Now**

- Never
- Less than once a week

Once a week

Twice a week

3 times a week

4 times a week

5 times a week

6 times a week

Every day

Several times each
day

### (160-161). How often did you/do you add salt to the food you eat?

**Before Now**

- Never
- Less than once a week

Once a week

Twice a week

3 times a week

4 times a week

5 times a week

6 times a week

Every day

Several times each
day

### (162-163). How often did you/do you eat industrially processed food for dinner? (freeze-dried instant food, og pre-cooked meals)

**Before Now**

- Never
- Less than once a week

Once a week

Twice a week

3 times a week

4 times a week

5 times a week

6 times a week

Every day

### (164-165). How often did you/do you eat so much that you are more than full (feel that you have eaten too much)?

**Before Now**

- Never
- Less than once a week

Once a week

Twice a week

3 times a week

4 times a week

5 times a week

6 times a week

Every day

Several times each
day

### (166-167). How often did you/do you eat candy, salty snacks or other unhealthy food even if you don’t think it tastes very good?

**Before Now**

- Never
- Less than once a week

Once a week

Twice a week

3 times a week

4 times a week

5 times a week

6 times a week

Every day

Several times each
day

### (168-169). When you buy groceries, how often did you/do you check the ingredient list?

**Before Now**

Never

Once in a while

Usually

Always

### Which size did you/do you usually choose when you buy:

Before you became pregnant:

**Large Small**

(171) Potato chips 350g 150g

(173) chocolate ≥80g <80g

(175) Soda 1,5l 0,5l

Now:

**Large Small**

(171) Potato chips 350g 150g

(173) chocolate ≥80g <80g

(175) Soda 1,5l 0,5l

In this last section, we would like you to mark what you ate and drank yesterday:

**Yes No**

176. Breakfast

177. Lunch

178. Dinner

179. Late supper

180. Snack

181. Whole milk

182. Low-fat milk

183. Skimmed milk

184. Juice

185. Fruit nectar

186. Soda/punch with sugar

187. Soda/punch without sugar

188. Beverages containing alcohol

189. Tap water

190. Bottled water, plain

191. Bottled water with carbonation
 or added flavor

192. Potatoes

193. Vegetables at dinner

194. Vegetables on a sandwich

195. Other vegetables (for example,
carrots at lunch)

196. Apple, orange,

pear or banana

197. Other fruits or berries

(other than apple, orange,
pear or banana)

198. Sweet buns (sweet rolls,
 “boller”, etc)

**220. If not, why not?**

199. Cake, muffins, etc.

200. Cereal without added sugar

201. Cereal containing sugar

202. Plain yogurt/yogurt
 without added sugar

203. Yogurt with added sugar

204. Instant noodles

(for example, Mr. Lee)

205. Potato chips/other salty snacks 206. Chocolate/other sweets 207. Hot dog from kiosk/gas station

208. French fries from fast-food chain

209. Industrially processed food, liked freeze-dried or pre-cooked food?

210. Did you add SUGAR to your
 food yesterday?

211. Did you add SALT to your
 food yesterday?

**Did you buy the following yesterday? (Make a mark for each food item)**

Yes No

(212) Potato chips

(213) Chocolate

(214) Soda

**If you answered “yes”, which size did you buy?**

Large Small

(215) Potato chips

(216) Chocolate

(217) Soda

(218) **Which day of the week was it yesterday?**

Monday

Tuesday

Wednesday

Thursday

Friday

Saturday

Sunday

(219) **Was yesterday a completely normal weekday?**

Yes No

Thank you for your help!
