# Supplementary material for: Study protocol: fit for delivery - can a lifestyle intervention in pregnancy result in measurable health benefits for mothers and newborns? A randomized controlled trial
Source: BMC Public Health. 2013 Feb 13;13:132. doi: 10.1186/1471-2458-13-132 (PMC3577450; doi:10.1186/1471-2458-13-132)
Supplement: Additional file 4 — The written, English version of the questionnaire which is completed at 6 months postpartum. [file 1471-2458-13-132-S4.docx]

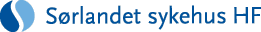


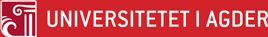
FitF

Fit for Delivery

Thank you for choosing to participate in our research study “Fit for Delivery”.
We wish to learn how you are doing now, about 1 year after delivery.

Please take about 20-30 minutes of your time and complete this survey. Read the questions carefully, and answer as best you can. Use black or blue ink, and make an “X” inside the box. Write clearly, where necessary.

Please write the date for completion of the survey in the box at the bottom of the page. The survey can then be delivered to your health care station at the time of your child’s 12 month examination, or mailed to us directly in a stamped, addressed envelope. In all cases, your answers will be treated confidentially—your answers can not be traced back to you.

**Thank you for your help!**

Sincerely,

The *Fit for Delivery* team

FFF 1 year postpartum

Date of completion:­­­­­­­­­­­­­__________________


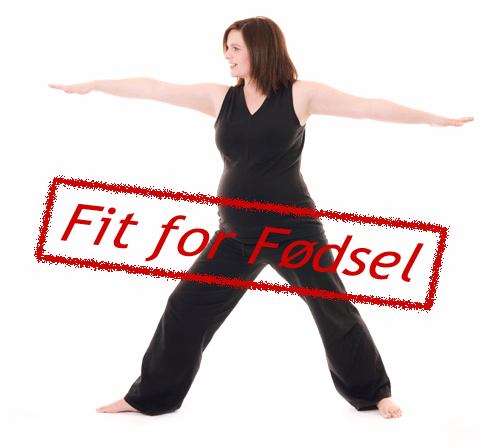


### 001. Participant number (please write clearly):

___________________

### 002. What is your date of birth?

___________________

### 003. How many months have gone since your delivery?

_______________ months

### 004. What is your primary activity?

Working outside the home

Student

Unemployed

Prolonged sick leave/disabled

Homemaker

Maternity leave

Part-time employment combined with part-time maternity leave

### 005. Are you currently breastfeeding?

No

Yes, exclusively breastfeeding

Yes, breastfeeding in addition to other food

### 006. How long did you breastfeed EXCLUSIVELY (the baby did not get anything other than breast milk, with the possible exception of vitamins)?

I never breastfed exclusively

­­­­­­­­­­­­­­___________ weeks

___________ months

### 007. How long have you breastfed the baby (either exclusively or in addition to formula, porridge, etc.)?

I never breastfed

­­­­­­­­­­­­­­___________ weeks

___________ months

008. **Do you smoke?**

Never smoked

Smoked before I became pregnant, but have stopped completely

Smoke 1-4 cigs / day

Smoke 5-9 cigs / day

Smoke 10-20 cigs / day

Smoke > 20 cigs / day

009. **Do you use snuff?**

Have never used snuff

Used snuff occasionally before I became pregnant, but have stopped completely

Used snuff regularly before I became pregnant, but have stopped completely

Use snuff occasionally

Use snuff daily, about

**010**. _____ doses per day

**011. Do you use any medication daily?**

No

Yes

**012. If yes, which? (Name of medication):**

___________________

___________________

**013. Do you use any vitamins or supplements daily?**

No

Yes

**014. If yes, which (name of supplement- iron, folate, etc.)?**

___________________

___________________

**015. Have you ever used any form of drugs/narcotics?**

Never tried

Used drugs regularly before I became pregnant, but have stopped completely

Have tried drugs in the past, but have stopped completely

Use drugs occasionally

Use drugs on a weekly basis

**016. If yes, which? (name of drug/ narcotic):**

___________________

___________________

### 017. How would you describe your own health? (choose one):

Very good

Good

Neither good nor bad

Poor

Very poor

**018. To what extent does your health limit your activities of daily life? (choose one):**

To a large extent

To some extent

Very little

Not at all

**019. If you are employed outside the home, have you had more than 1 week of sick leave during the past month ?**

Yes

No

020. **If yes, how long have you had sick leave? (choose the answer that fits best):**

1-2 weeks, partial sick leave

1-2 weeks, complete sick leave

2-3 weeks, partial sick leave

2-3 weeks, complete sick leave

3-4 weeks, partial sick leave

3-4 weeks, complete sick leave

4+ weeks, partial sick leave

4+ weeks, complete sick leave

Physical activity

We would now like to ask you about the physical activities you do. We are interested in information about different kinds of physical activity that are part of women’s daily lives. Please answer all questions, regardless of how active you believe yourself to be. Think of activities you do at work, as part of your house and yard work, to get from place to place, and in your spare time (for recreation, exercise or sport).

Think of all **vigorous** physical activities you have performed over the last 7 days. Vigorous physical activities refer to activities that take hard physical effort and make you breathe much harder than normal. Include only those activities that last for at least 10 minutes at a time.

**021. During the last 7 days, on how many days did you do vigorous physical activities like heavy lifting, digging, aerobics or fast biking?**

_____ days

No vigorous physical activities: *Go to question 23.*

**022. How much time did you usually spend on one of those days doing vigorous physical activities?**

0. Don’t know

1. 10 minutes

2. 20 minutes

3. 30 minutes

4. 40 minutes

5. 50 minutes

6. 1 hour

7. 1 hour and 10 minutes

8. 1 hour and 20 minutes

9. 1 hour and 30 minutes

10. 1 hour and 40 minutes

11. 1 hour and 50 minutes

12. 2 hours or more

Think of all **moderate** physical activities you have done over the last 7 days. Moderate physical activities are activities that take moderate physical effort and make you breathe somewhat harder than normal. Include only those activities that last for at least 10 minutes at a time.

**023. During the last 7 days, on how many days did you do moderate physical activities like carrying light loads, bicycling at a regular pace, or light jogging? Do not include walking.**

_____ days

No moderate physical activities: *Go to question 25.*

**024. How much time did you usually spend on one of those days doing moderate physical activities?**

0. Don’t know

1. 10 minutes

2. 20 minutes

3. 30 minutes

4. 40 minutes

5. 50 minutes

6. 1 hour

7. 1 hour and 10 minutes

8. 1 hour and 20 minutes

9. 1 hour and 30 minutes

10. 1 hour and 40 minutes

11. 1 hour and 50 minutes

12. 2 hours or more

Think about the time you have spent walking during the last 7 days. This includes walking at work and at home, walking to travel from place to place, and any other walking that you did solely for recreation, sport, exercise or leisure.

**025.** During the last 7 days, on how many days did you walk for at least 10 minutes at a time?

_____ days

Didn’t walk: *Go to question 27.*

**026. How much time in total did you usually spend walking on one of those days?**

0. Don’t know

1. 10 minutes

2. 20 minutes

3. 30 minutes

4. 40 minutes

5. 50 minutes

6. 1 hour

7. 1 hour and 10 minutes

8. 1 hour and 20 minutes

9. 1 hour and 30 minutes

10. 1 hour and 40 minutes

11. 1 hour and 50 minutes

12. 2 hours or more

The next question is about the time you spent sitting on weekdays while at work, at home, while doing course work and during leisure time. This includes time spent sitting at a desk, visiting friends, reading, traveling on a bus or sitting or lying down to watch television.

**027. During the last 7 days, how much time in total did you usually spend sitting on a week day?**

Answer: _______ hours

### How do you usually get to work/school?

**(044):**

Walk

Bike

Public transportation (bus, train, etc.)

Car

Motorcycle, scooter or moped

Not applicable (not working, going to school)

**Below you will find a list of reasons for NOT doing physical activities.** Please mark one or more boxes for the reason(s) that are most important for you:

**(065)** Don’t have the time

**(066)** Can’t afford it

**(067)** Transportation problems

**(068)** Negative experiences

**(069)** Problems with mobility

**(070)** Don’t think I can do it

**(071)** Don’t have the energy

**(072)** Afraid to get hurt (to fall, get a sprain)

**(073)** Would rather use my time on other things

**(074)** Because of my physical health

**(075)** Don’t have anyone to do physical activities with me

**(076)** Schedules don’t fit for me

**(077)** Don’t know of anything available to me

**(078)** Afraid to go out

**(079)** Nothing available in my area of interest

**(081)** Fear of urinary incontinence

**(083)** Pelvic pain

**(085)** Other reasons

If you have other reasons, please explain:

**085.** _______________________________________________________

_______________________________________________________

What do you usually eat?

When you answer these questions, think about what you usually eat. Consider what you eat at home, at work, and in your spare time. Mark the box that you feel best fits for you.

### 087. How often do you eat breakfast?

- Never
- Less than once a week

Once a week

Twice a week

3 times a week

4 times a week

5 times a week

6 times a week

Every day

**(089). How often do you eat lunch?**

- Never
- Less than once a week

Once a week

Twice a week

3 times a week

4 times a week

5 times a week

6 times a week

Every day

### (091). How often do you eat dinner?

- Never
- Less than once a week

Once a week

Twice a week

3 times a week

4 times a week

5 times a week

6 times a week

Every day

### (093). How often do you eat a late supper (kveldsmat)?

- Never
- Less than once a week

Once a week

Twice a week

3 times a week

4 times a week

5 times a week

6 times a week

Every day

### (095). How often do you eat snacks?

- Never
- Less than once a week

Once a week

Twice a week

3 times a week

4 times a week

5 times a week

6 times a week

Every day

- Several times each day

### (097). How often do you drink whole milk?

- Never
- Less than once a week

Once a week

Twice a week

3 times a week

4 times a week

5 times a week

6 times a week

Every day

- Several times each day

### (099). How often do you drink low-fat milk?

- Never
- Less than once a week

Once a week

Twice a week

3 times a week

4 times a week

5 times a week

6 times a week

Every day

- Several times each day

### (101). How often do you drink skimmed milk?

- Never
- Less than once a week

Once a week

Twice a week

3 times a week

4 times a week

5 times a week

6 times a week

Every day

- Several times each day

### (103). How often do you drink juice?

- Never
- Less than once a week

Once a week

Twice a week

3 times a week

4 times a week

5 times a week

6 times a week

Every day

- Several times each day

### (105). How often do you drink fruit nectar?

- Never
- Less than once a week

Once a week

Twice a week

3 times a week

4 times a week

5 times a week

6 times a week

Every day

- Several times each day

### (107). How often do you drink soda/soft drinks – *with sugar*?

- Never
- Less than once a week

Once a week

Twice a week

3 times a week

4 times a week

5 times a week

6 times a week

Every day

- Several times each day

### (109). How often do you drink soda/soft drinks—*without sugar*?

- Never
- Less than once a week

Once a week

Twice a week

3 times a week

4 times a week

5 times a week

6 times a week

Every day

- Several times each day

### (111). How often do you drink beverages that contain alcohol?

- Never
- Less than once a week

Once a week

Twice a week

3 times a week

4 times a week

5 times a week

6 times a week

Every day

- Several times each day

### (113). How often do you drink tap water?

- Never
- Less than once a week

Once a week

Twice a week

3 times a week

4 times a week

5 times a week

6 times a week

Every day

- Several times each day

### (115). How often do you drink bottled water (without carbonation or flavor added)?

- Never
- Less than once a week

Once a week

Twice a week

3 times a week

4 times a week

5 times a week

6 times a week

Every day

- Several times each day

### (117). How often do you drink bottled water with carbonation or flavor added?

- Never
- Less than once a week

Once a week

Twice a week

3 times a week

4 times a week

5 times a week

6 times a week

Every day

- Several times each day

### (119). How often do you drink coffee?

- Never
- Less than once a week

Once a week

Twice a week

3 times a week

4 times a week

5 times a week

6 times a week

Every day

- Several times each day

### (121). How often do you eat potatoes?

- Never
- Less than once a week

Once a week

Twice a week

3 times a week

4 times a week

5 times a week

6 times a week

Every day

- Several times each day

### (123). How often do you eat vegetables at dinner?

- Never
- Less than once a week

Once a week

Twice a week

3 times a week

4 times a week

5 times a week

6 times a week

Every day

### (125). How often do you eat vegetables on your sandwich?

- Never
- Less than once a week

Once a week

Twice a week

3 times a week

4 times a week

5 times a week

6 times a week

Every day

- Several times each day

### (127). How often do you eat other vegetables (for example, carrots at lunchtime)?

- Never
- Less than once a week

Once a week

Twice a week

3 times a week

4 times a week

5 times a week

6 times a week

Every day

- Several times each day

### (129). How often do you eat apples, oranges, pears or bananas?

- Never
- Less than once a week

Once a week

Twice a week

3 times a week

4 times a week

5 times a week

6 times a week

Every day

- Several times each day

### (131). How often do you eat other fruits or berries (fruits or berries other than apples, oranges, pears or bananas)?

- Never
- Less than once a week

Once a week

Twice a week

3 times a week

4 times a week

5 times a week

6 times a week

Every day

- Several times each day

### (133). How often do you eat fruits or vegetables as snacks?

- Never
- Less than once a week

Once a week

Twice a week

3 times a week

4 times a week

5 times a week

6 times a week

Every day

- Several times each day

### (135). How often do you eat cookies or crackers?

- Never
- Less than once a week

Once a week

Twice a week

3 times a week

4 times a week

5 times a week

6 times a week

Every day

- Several times each day

### (137). How often do you eat sweet buns (sweet rolls, “boller”, etc)?

- Never
- Less than once a week

Once a week

Twice a week

3 times a week

4 times a week

5 times a week

6 times a week

Every day

- Several times each day

### (139). How often do you eat cake, muffins, etc.?

- Never
- Less than once a week

Once a week

Twice a week

3 times a week

4 times a week

5 times a week

6 times a week

Every day

- Several times each day

### (141). How often do you eat cereal without added sugar?

- Never
- Less than once a week

Once a week

Twice a week

3 times a week

4 times a week

5 times a week

6 times a week

Every day

- Several times each day

### (143). How often do you eat cereal containing sugar?

- Never
- Less than once a week

Once a week

Twice a week

3 times a week

4 times a week

5 times a week

6 times a week

Every day

- Several times each day

### (145). How often do you eat plain yogurt (yogurt without added sugar)?

- Never
- Less than once a week

Once a week

Twice a week

3 times a week

4 times a week

5 times a week

6 times a week

Every day

- Several times each day

### (147). How often do you eat yogurt with added sugar?

- Never
- Less than once a week

Once a week

Twice a week

3 times a week

4 times a week

5 times a week

6 times a week

Every day

- Several times each day

### (149). How often do you eat instant noodles (for example, Mr. Lee)?

- Never
- Less than once a week

Once a week

Twice a week

3 times a week

4 times a week

5 times a week

6 times a week

Every day

- Several times each day

### (151). How often do you eat potato chips/other salty snacks?

- Never
- Less than once a week

Once a week

Twice a week

3 times a week

4 times a week

5 times a week

6 times a week

Every day

- Several times each day

### (153). How often do you eat chocolate/ other sweets?

- Never
- Less than once a week

Once a week

Twice a week

3 times a week

4 times a week

5 times a week

6 times a week

Every day

- Several times each day

### (155). How often do you eat hot dogs/ sausages from a gas station or kiosk?

- Never
- Less than once a week

Once a week

Twice a week

3 times a week

4 times a week

5 times a week

6 times a week

Every day

- Several times each day

### (157). How often do you eat french fries from a fast-food chain?

- Never
- Less than once a week

Once a week

Twice a week

3 times a week

4 times a week

5 times a week

6 times a week

Every day

- Several times each day

### (159). How often do you add sugar to the food you eat?

- Never
- Less than once a week

Once a week

Twice a week

3 times a week

4 times a week

5 times a week

6 times a week

Every day

- Several times each day

### (161). How often do you add salt to the food you eat?

- Never
- Less than once a week

Once a week

Twice a week

3 times a week

4 times a week

5 times a week

6 times a week

Every day

- Several times each day

### (163). How often do you eat industrially processed food for dinner? (freeze-dried instant food, or pre-cooked meals)

- Never
- Less than once a week

Once a week

Twice a week

3 times a week

4 times a week

5 times a week

6 times a week

Every day

### (165). How often do you eat so much that you are more than full (feel that you have eaten too much)?

- Never
- Less than once a week

Once a week

Twice a week

3 times a week

4 times a week

5 times a week

6 times a week

Every day

- Several times each day

### (167). How often do you eat candy, salty snacks or other unhealthy food even if you don’t think it tastes very good?

- Never
- Less than once a week

Once a week

Twice a week

3 times a week

4 times a week

5 times a week

6 times a week

Every day

- Several times each day

### (169). When you buy groceries, how often do you check the ingredient list?

Never

Once in a while

Usually

Always

### Which size do you usually choose when you buy:

**Large Small**

(171) Potato chips 350g 150g

(173) chocolate ≥80g <80g

(175) Soda 1,5l 0,5l

In this last section, we would like you to mark what you ate and drank yesterday:

**Yes No**

176. Breakfast

177. Lunch

178. Dinner

179. Late supper

180. Snack

181. Whole milk

182. Low-fat milk

183. Skimmed milk

184. Juice

185. Fruit nectar

186. Soda/punch with sugar

187. Soda/punch without sugar

188. Beverages containing alcohol

189. Tap water

190. Bottled water, plain

191. Bottled water with carbonation
 or added flavor

192. Potatoes

193. Vegetables at dinner

194. Vegetables on a sandwich

195. Other vegetables (for example,
carrots at lunch)

196. Apple, orange,

pear or banana

197. Other fruits or berries

(other than apple, orange,
pear or banana)

198. Sweet buns (sweet rolls,
 “boller”, etc)

**220. If not, why not?**

199. Cake, muffins, etc.

200. Cereal without added sugar

201. Cereal containing sugar

202. Plain yogurt/yogurt
 without added sugar

203. Yogurt with added sugar

204. Instant noodles

(for example, Mr. Lee)

205. Potato chips/other salty snacks 206. Chocolate/other sweets 207. Hot dog from kiosk/gas station

208. French fries from fast-food chain

209. Industrially processed food, liked freeze-dried or pre-cooked food?

210. Did you add SUGAR to your
 food yesterday?

211. Did you add SALT to your
 food yesterday?

Thank you for your help!

**Did you buy the following yesterday? (Make a mark for each food item)**

Yes No

(212) Potato chips

(213) Chocolate

(214) Soda

**If you answered “yes”, which size did you buy?**

Large Small

(215) Potato chips

(216) Chocolate

(217) Soda

(218) **Which day of the week was it yesterday?**

Monday

Tuesday

Wednesday

Thursday

Friday

Saturday

Sunday

(219) **Was yesterday a completely normal weekday?**

Yes No
